# Comparing Nipple and Areola Sensory Outcomes and Nerve-Related Complications Using Different Incision Types in Breast Augmentation: A Scoping Review

**DOI:** 10.1007/s00266-026-05681-x

**Published:** 2026-02-18

**Authors:** Bisher Tulimat, Yehia Elshafey, Omar Kiwan

**Affiliations:** 1 Royal College of Surgeons in Ireland, Medical University of Bahrain, Busaiteen, Bahrain; 2 Faculty of Biology, Medicine and Health, The University of Manchester, Manchester, UK

**Keywords:** Breast augmentation, Nipple–areola complex, Sensory outcomes, Incision type, Nerve injury, Aesthetic breast surgery

## Abstract

**Background:**

Changes in nipple–areola complex sensation are a major concern after breast augmentation. Multiple incision options exist such as inframammary, periareolar, transareolar, transaxillary, and transumbilical, but their sensory impact remains unclear. This scoping review synthesizes evidence on incision type, nipple–areola outcomes, and nerve-related complications.

**Methods:**

Following PRISMA-ScR, PubMed, Embase, Scopus, Cochrane, and Google Scholar were searched to July 2025. Eligible studies reported sensory outcomes by incision type. Both subjective and objective measures were included.

**Results:**

Thirty-seven studies were included, encompassing over 13,000 patients across primary clinical cohorts, with several large meta-analyses contributing additional pooled data. Most studies evaluated inframammary and periareolar incisions. Persistent nipple sensory change was generally low (<5%) but varied with incision and methodology. Large cohorts showed periareolar incisions had ~3× higher risk of hypoesthesia and pain than inframammary. Prospective studies often found no difference, though inframammary incisions could impair lower-pole sensation. Lateralized inframammary incisions preserved nipple–areola complex sensitivity, unlike central cuts. Transareolar incisions caused mild objective hyposensitivity but high subjective satisfaction. Transaxillary data were limited but anatomically suggest higher risk. Transumbilical consistently showed no nipple–areola complex changes. Heterogeneity in outcome definitions, testing tools, and follow-up limited comparisons.

**Conclusions:**

Incision type influences nipple sensation but is modified by technique and implant plane. Periareolar incisions show higher risk in large cohorts, while lateralized inframammary access may be protective. Transumbilical avoids breast sensory risk but is device limited. Evidence for transaxillary and perinipple approaches is sparse. Standardized, objective testing is needed to guide counseling and surgical planning.

**Level of Evidence V:**

This journal requires that authors assign a level of evidence to each article. For a full description of these Evidence-Based Medicine ratings, please refer to the Table of Contents or the online Instructions to Authors www.springer.com/00266

**Supplementary Information:**

The online version contains supplementary material available at 10.1007/s00266-026-05681-x.

## Introduction

Breast augmentation remains one of the most frequently performed aesthetic procedures worldwide [1]. Alongside aesthetic outcomes, preservation of nipple–areola complex (NAC) sensation is a critical concern for both patients and surgeons. Sensory changes following augmentation can influence not only quality of life and sexual well-being but also functional outcomes such as breastfeeding [2]. A large systematic review estimated that nerve injury or sensory disturbance occurs in approximately 14–15% of patients after breast augmentation, underscoring the clinical relevance of incision related sensory risk [3].

NAC innervation arises primarily from the lateral cutaneous branch of the fourth intercostal nerve with contributions from adjacent intercostals and anterior cutaneous branches [4]. Common approaches including inframammary, periareolar, transareolar, transaxillary, and transumbilical differ in exposure and scar location but traverse distinct neurovascular territories. Inframammary access typically avoids terminal anterior cutaneous branches [5], whereas periareolar incisions cross the areolar border where these branches converge [4]. Remote approaches such as axillary or umbilical incisions avoid breast scars but may endanger lateral sensory branches or face technical limitations [6, 7]. Despite extensive clinical use, evidence remains mixed on whether incision choice consistently predicts long-term NAC sensory outcomes. This heterogeneity complicates counseling, as fear of persistent nipple numbness or pain shapes choices [8]; thus, scar placement must be balanced against sensory risk.

The purpose of this scoping review is to map and synthesize existing evidence on NAC sensory outcomes and nerve-related complications following primary breast augmentation. This review aims to identify patterns, explore modifying factors such as implant plane and testing methodology, and highlight gaps requiring further investigation.

## Methods

This scoping review was conducted according to a pre-specified protocol developed in line with the PRISMA-ScR checklist. The methodology followed the Joanna Briggs Institute framework for scoping reviews, including the definition of the population, concept, and context (PCC) criteria, systematic searching, independent screening, data extraction, and synthesis.

### Eligibility Criteria

Studies were eligible if they included women undergoing primary cosmetic breast augmentation, reported outcomes on nipple–areola complex (NAC) sensory changes or nerve-related complications, and stratified or described outcomes by incision type. Both objective assessments (e.g., Semmes–Weinstein monofilaments, pressure-specified sensory devices, two-point discrimination) and subjective assessments (patient-reported hypoesthesia, paresthesia, hyperesthesia, pain) were included. Studies were restricted to English-language publications from January 2000 onwards. Exclusion criteria were secondary augmentations, revision surgeries, mastopexy–augmentation, reconstructive procedures, concurrent breast operations, and non-English publications.

### Information Sources and Search Strategy

A comprehensive search was performed on July 31, 2025, in PubMed, Embase, Scopus, and the Cochrane Library. Supplementary searches were conducted in Google Scholar to capture additional articles. The search strategy combined controlled vocabulary and keywords for breast augmentation, incision types (periareolar, inframammary, transareolar, transaxillary, transumbilical), and sensation or nerve injury. The full search strategies for each database are provided in Supplementary Appendix 1.

### Selection of Sources of Evidence

All records were imported into reference management software and duplicates were removed. Two reviewers independently screened titles/abstracts and full texts against eligibility criteria. Disagreements were resolved by discussion or arbitration between the reviewers.

### Data Charting Process

A standardized data-charting form was developed and applied to all included studies. Data extracted included study design, country, sample size, incision type, implant plane, implant type, follow-up, method of sensory assessment, and reported NAC outcomes. Data extraction was conducted in duplicate by two independent reviewers.

### Synthesis of Results

Findings were collated and presented in tabular and narrative form. Incision types were pre-specified and grouped as inframammary (central vs lateralized), periareolar (circumareolar, medial periareolar, broken-line), transareolar, perinipple, transaxillary, and transumbilical. Both objective and subjective outcomes were summarized. Methodological differences (e.g., implant plane, device type, testing tools) were considered in the interpretation.

## Results

### Study Selection

**Fig. 1:**
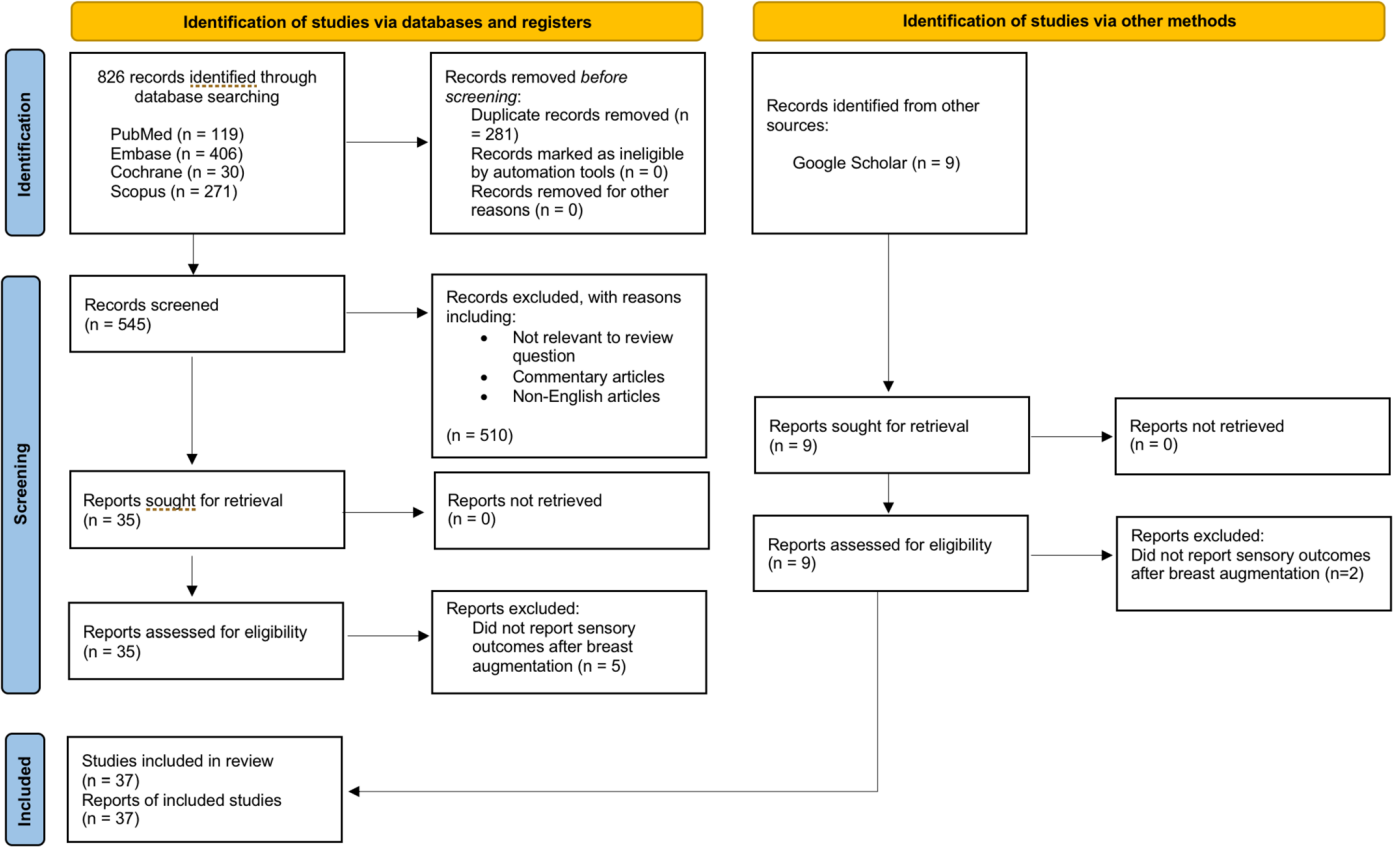
PRISMA flow diagram of study selection. Flow diagram illustrating the identification, screening, eligibility, and inclusion of studies according to the PRISMA-ScR guidelines. The diagram summarizes the number of records identified from each database, duplicates removed, and the final number of studies included in the review (*n* = 37)

The search strategy identified 826 records (PubMed 119, Embase 406, Cochrane 30, Scopus 271), with nine additional articles from Google Scholar. After removal of 281 duplicates, 545 records were screened by title and abstract. Following full-text review with eligibility criteria applied, 37 articles published from 2000 onward were included. Exclusions were primarily due to irrelevant outcomes, lack of incision-specific data, or secondary/revision surgery populations. The PRISMA flow diagram is shown in Fig. 1.

### Characteristics of Included Studies

The 37 included studies encompassed a wide spectrum of designs. Three were systematic reviews and meta-analyses [3, 9, 10], and two were narrative reviews [11, 12]. Among the clinical studies, there were ten prospective cohorts [13–22], fourteen retrospective cohorts or case series [23–36], three large retrospective registry/series analyses [37–39], two cross-sectional surveys [40, 41], and one cadaveric anatomical study [42]. In addition, two studies used hybrid designs, one combining a retrospective cohort and cadaveric anatomical analysis [43] and one combining a cross-sectional survey and retrospective chart review [44]. The complete dataset with extended study characteristics, including patient demographics, implant variables, and sensory assessment methods, is available in Supplementary Appendix 2.

Sample sizes ranged widely, from small case series of fewer than 20 patients [22, 27, 35, 36] to large datasets exceeding 1,000 patients [16, 23, 34, 37, 39, 40]. The largest pooled analysis synthesized outcomes in over 25,000 patients [10].

Follow-up durations also varied considerably. Short-term cohorts (≤6 months) focused on early recovery of NAC sensibility [13, 14, 18–20, 23], while medium-term studies (6–24 months) offered intermediate perspectives [15, 22, 24, 27, 35, 36]. Long-term follow-up (>24 months) was provided in multiple series and registry-based analyses [16, 17, 21, 26, 28–30, 32–34, 37–40, 44]. Reviews and meta-analyses encompassed variable follow-up across included studies. Overall, long-term follow-up was the most frequently reported, particularly in large retrospective series and registry analyses.

Geographically, the included studies were conducted across a wide range of countries. TheUSA contributed the largest number (*n*=10), followed by Brazil (*n*=3), Turkey (*n*=3), China (*n*=2), Korea (*n*=2), Egypt (*n*=2), Germany (*n*=1), Puerto Rico (*n*=1), Argentina (*n*=1), Sweden (*n*=1), Singapore (*n*=1), Australia (*n*=1), Taiwan (*n*=1), Italy (*n*=1), Switzerland (*n*=1), and Denmark (*n*=1). In addition, five reviews included multi-country datasets [3, 9–12]. A summary of study characteristics, including design, sample size, follow-up, geography, and methods of assessment, is shown in Table 1.

**Table 1:** Summary of study characteristics of included articles (*n*=37)

Authors	Country	Design	*N* (patients)	Follow-up duration	Incision types	Method of assessment
Chen et al. [9]	International (multi-country)	Systematic review & meta-analysis	9,965	Variable (across studies)	Inframammary, periareolar, transaxillary, transumbilical	NA
Shi et al. [30]	China	Retrospective cohort (comparative)	144	Long (>24 mo)	Periareolar, transaxillary	Subjective
Ducic, Seiboth and Lorio [43]	USA	Retrospective cohort & cadaveric anatomical study	57	NR	Periareolar, inframammary, transaxillary, transumbilical	Semi-objective
Cruz and Korchin [25]	Puerto Rico	Retrospective cohort (comparative)	105	NR	Periareolar, inframammary	Subjective
Rancati et al. [20]	Argentina	Prospective cohort (comparative)	50	Short (≤6 mo)	Inframammary (medial & lateral variants)	Objective
Ashraf et al. [13]	Egypt	Prospective cohort (comparative)	18	Short (≤6 mo)	Inframammary	Objective
Wieslander [34]	Sweden	Retrospective case series	1,130	Long (>24 mo)	Transaxillary	Subjective
Frascino and Pompei [26]	Brazil	Retrospective case series	32	Long (>24 mo)	Periareolar (perinipple)	Subjective
Shen et al. [10]	International (multi-country)	Systematic review & Bayesian network meta-analysis	25,744	Variable (across studies)	Inframammary, periareolar, transaxillary	Subjective
Ballard et al. [40]	USA	Cross-sectional study	11,756	Long (>24 mo)	Inframammary, periareolar, transaxillary, transumbilical	Subjective
Randquist et al. [29]	Singapore	Retrospective case series	22	Long (>24 mo)	Inframammary	Subjective
Brown [14]	Australia	Prospective cohort	162	Short (≤6 mo)	Inframammary	Objective
Lund et al. [16]	USA	Prospective cohort	4927	Long (>24 mo)	Inframammary, periareolar	Subjective
Zelken et al. [35]	Taiwan	Retrospective case series	11	Medium (6-24 mo)	Transareolar (TAPA, zigzag variant)	Subjective
Zhu et al. [36]	China	Retrospective case series	15	Medium (6-24 mo)	Periareolar (infra-nipple broken line incision)	Subjective
Ducic et al. [3]	International (multi-country)	Systematic review & meta-analysis	8361	Variable (across studies)	Inframammary, periareolar, transaxillary	Mixed
Swanson [21]	USA	Prospective cohort	225	Long (>24 mo)	Inframammary, periareolar, transaxillary, transnipple	Subjective
Aygit, Basaran and Mercan [24]	Turkey	Prospective case series	27	Medium (6-24 mo)	Transaxillary	Subjective
Araco et al. [23]	Italy	Retrospective cohort (comparative)	1222	Short (≤6 mo)	Periareolar, inframammary	Subjective
Reece et al. [41]	USA	Cross-sectional survey	NA (surgeons surveyed)	NA	Inframammary, periareolar, transaxillary, transumbilical	Subjective
Hammond [11]	International (multi-country)	Narrative review	NA	NA	Periareolar	NA
Tenius, Freitas and Ono [22]	Brazil	Prospective case series	10	Medium (6-24 mo)	Transareolar (zigzag)	Subjective
Pitanguy et al. [19]	Brazil	Prospective cohort	29	Short (≤6 mo)	Periareolar, inframammary, transareolar	Mixed
Michalopoulos [12]	International (multi-country)	Narrative review	NA	NA	Periareolar, inframammary, transaxillary, transumbilical	NA
Mofid et al. [17]	USA	Prospective cohort (comparative)	20	Long (>24 mo)	Periareolar, inframammary	Objective
Okwueze et al. [18]	USA	Prospective cohort (comparative)	33	Short (≤6 mo)	Periareolar, inframammary	Mixed
Stoff-Khalili et al. [32]	Germany	Retrospective cohort (comparative)	328	Long (>24 mo)	Periareolar	Subjective
Hwang et al. [42]	Korea	Cadaveric anatomical study	NA (cadaveric study)	NA	NA	NA
Kompatscher, Schuler and Beer [28]	Switzerland	Retrospective cohort	18	Long (>24 mo)	Transareolar	Mixed
Karabulut et al. [27]	Turkey	Retrospective case series	5	Medium (6-24 mo)	Periareolar (medial)	Subjective
Sohn et al. [31]	Korea	Retrospective case series	19	NR	Periareolar	Subjective
Caleel [38]	USA	Retrospective case series	653	Long (>24 mo)	Transumbilical	Subjective
Helmy [15]	Egypt	Prospective cohort (comparative)	30	Medium (6-24 mo)	Periareolar, inframammary	Mixed
Huang, Wichmann and Mills [39]	USA	Retrospective case series	1,682	Long (>24 mo)	Transaxillary	NA
Akkary et al. [37]	USA	Retrospective case series	2263	Long (>24 mo)	Transumbilical	NA
Üstün et al. [33]	Turkey	Retrospective cohort (comparative)	55	Long (>24 mo)	Inframammary	Objective
Sperling et al. [44]	Denmark	Cross-sectional survey + retrospective chart review	95	Long (>24 mo)	Inframammary	Subjective

### Methods of Sensory Assessment

**Fig. 2:**
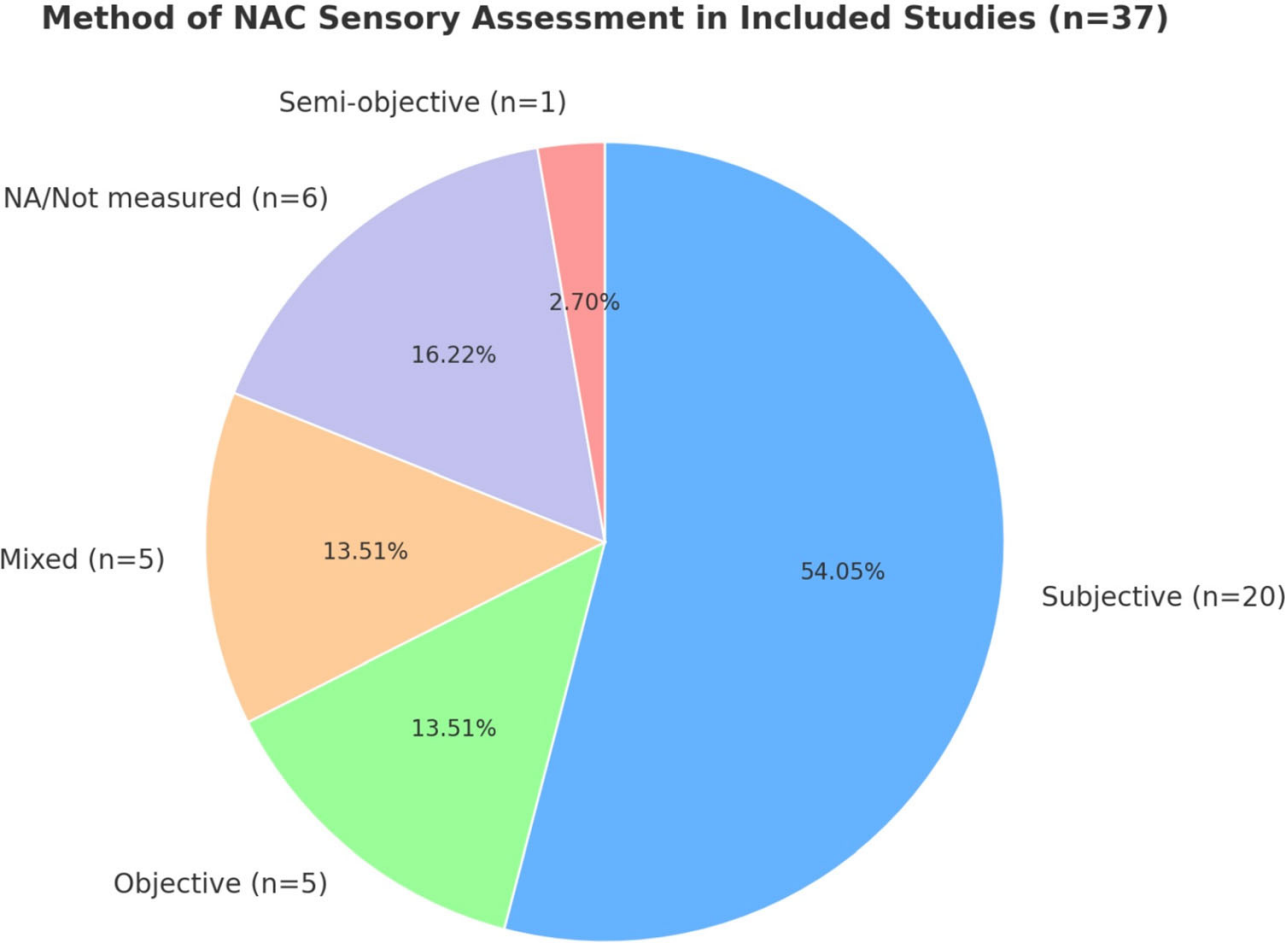
Methods of nipple–areola complex (NAC) sensory assessment in included studies (*n* = 37). Pie chart illustrating the types of sensory assessment methods reported across included studies, categorized as subjective, objective, mixed, semi-objective, or unspecified

Approaches to measuring nipple–areola complex (NAC) sensation were highly variable. Twenty studies (54.1%) relied solely on subjective patient-reported outcomes, while five (13.5%) used purely objective measures such as Semmes–Weinstein monofilaments or pressure-specified sensory devices. Five studies (13.5%) used mixed methods combining subjective and objective measures, one (2.7%) used semi-objective cadaveric/clinical correlation. In six studies (16.2%), sensation outcomes were either mentioned without describing the assessment method, only reported in passing, or not formally measured at all (Fig. 2).

### Distribution of Incision Types

**Fig. 3:**
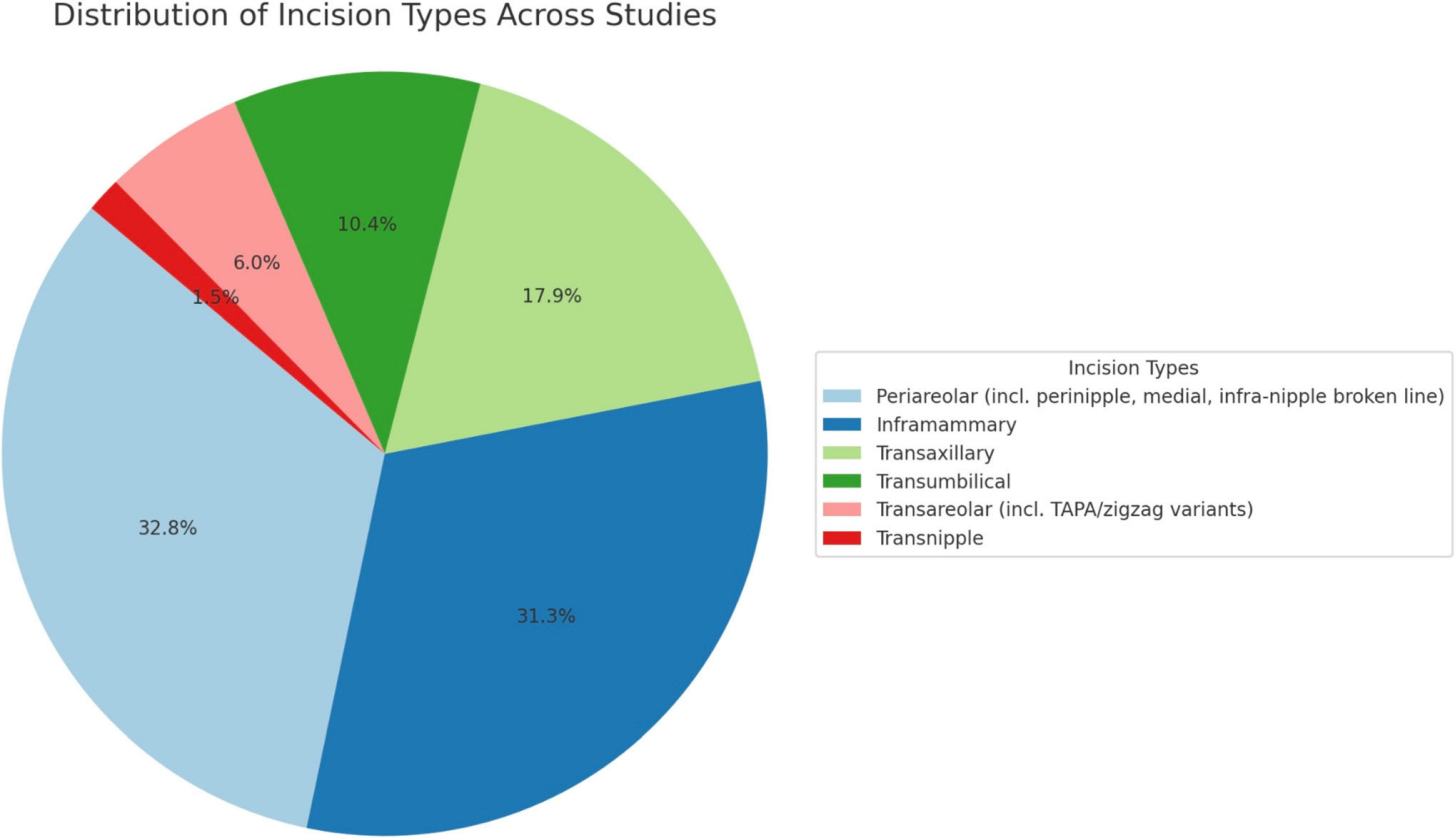
Distribution of incision types across studies (per-study counts; see legend for grouped variants). Pie chart illustrating the proportion of incision types reported across all included studies, grouped as inframammary, periareolar, transaxillary, transumbilical, and transareolar approaches, with variants (e.g., medial or perinipple) classified within their respective categories

Across the 37 studies, periareolar incisions (including medial, perinipple, and broken-line variants) were reported in 32.8%, inframammary in 31.3%, transaxillary in 17.9%, transumbilical in 10.4%, transareolar in 6%, and transnipple in 1.5% (Fig. 3). Together, periareolar and inframammary incisions accounted for nearly two-thirds of the published evidence (64.1%).

### Sensory Outcomes by Incision Type

#### Inframammary Fold Incisions

The inframammary fold (IMF) incision was one of the most frequently studied. It was reported in 21 articles either alone or alongside other approaches.

Prospective quantitative studies demonstrated variable outcomes depending on incision placement and implant plane. Central IMF incisions were associated with markedly higher rates of persistent NAC sensitivity loss, whereas lateralized IMF incisions consistently preserved full sensation [20]. Other prospective assessments using objective devices found no overall difference in NAC sensitivity between IMF and periareolar incisions, although IMF access demonstrated greater sensory loss in the inferior breast [18]. In one prospective series, sensitivity loss was observed after both IMF and periareolar augmentations, but this effect was driven primarily by implant-to-breast volume ratio rather than incision type [19].

Retrospective data reinforced the generally low rates of persistent sensory change with IMF incisions. In small cohorts, only isolated cases of persistent mild hypoesthesia were reported at one year [15], and in larger series transient postoperative numbness was common but long-term persistent sensory loss remained infrequent, occurring in only a small minority of patients [21].

In contrast, some longer-term series have reported much higher overall rates of breast sensory change and persistent pain. However, these outcomes were not NAC-specific and may reflect broader breast sensation and pain syndromes [44].

Objective long-term studies provide further context. Using Semmes–Weinstein monofilaments, one study showed worse sensitivity in IMF-augmented patients versus controls, with subglandular placement preserving central NAC sensitivity but worsening peripheral sensation compared with dual-plane techniques [33].

Overall, IMF incisions showed low persistent NAC change, though central placement carried greater risk than lateralized incisions.

#### Periareolar Incisions

The periareolar approach was examined in 22 of the included studies, either as a primary incision or in comparison with inframammary and other access routes.

Large retrospective data suggest a higher risk of persistent NAC sensory change and pain with periareolar incisions, with one study of 1,222 primary augmentations reporting nearly a threefold increase in sensory alterations and more than a threefold increase in areolar pain compared with inframammary incisions [23].

Prospective quantitative studies provide a more nuanced picture. Across cohorts using pressure-specified sensory devices and other objective tools, NAC thresholds did not differ significantly between periareolar and inframammary incisions at short- to mid-term follow-up, while implant size and implant-to-breast volume ratio emerged as stronger predictors of altered sensitivity [17–19].

Smaller prospective series reinforce that persistent NAC changes are uncommon. Transient numbness was reported in a minority of periareolar cases early postoperatively, with resolution by one year in all patients, whereas an isolated persistent deficit was noted in an inframammary case [15]. Retrospective analyses of periareolar augmentations further suggest that implant plane may modify risk, with higher rates of altered nipple sensation in subglandular pockets compared with subpectoral or subfascial placements [32].

Technical refinements of the periareolar approach have also been described, with some authors emphasizing techniques that preserve superior intercostal nerve branches. When meticulous dissection is performed, these refinements suggest that sensory outcomes may be comparable to those of inframammary access [11]. Several small technical variants of periareolar access are described in the literature but are addressed separately under Sect. “Other Incision Variants.”

Taken together, the evidence for periareolar incisions is mixed. Large retrospective data strongly implicate the approach as a risk factor for sensory loss and pain [23] while prospective controlled studies with objective testing often report no significant difference compared with inframammary incisions [17–19]. Overall, absolute rates of persistent deficits appear low, but relative risk may be elevated compared with lateralized IMF incisions.

#### Transareolar Incisions

The transareolar approach was reported in only a few small studies, but those available included detailed sensory assessments. Objective testing using Semmes–Weinstein monofilaments, vibration, and temperature discrimination consistently demonstrated mild to moderate long-term NAC sensory deficits after transareolar access. However, subjective symptoms were far less pronounced, with only about one-fifth of patients reporting noticeable nipple changes, highlighting a clear discordance between objective and subjective measures [28]. Another small prospective cohort included transareolar cases but the sample was too small for valid statistical comparison, and no incision-specific conclusions could be drawn [19]. Collectively, these findings suggest that transareolar access can result in measurable sensory deficits on objective testing, though most patients remain satisfied subjectively [28].

#### Transaxillary Incisions

The transaxillary approach was represented in twelve studies, including large retrospective series, smaller clinical cohorts, and anatomic cadaveric dissections.

The largest series, spanning two decades and over 1,600 transaxillary subpectoral augmentations, reported no cases of nipple sensation loss. However, outcomes were based on chart review rather than objective testing [39]. Smaller clinical series similarly described “satisfactory” nipple sensitivity in all patients, but these reports relied entirely on subjective assessment [24].

In contrast, an anatomical cadaveric study demonstrated a clear vulnerability of the lateral cutaneous branch of the fourth intercostal nerve within the axillary tunnel, identifying a predictable “danger zone” that could predispose to NAC sensory loss [42].

A systematic review highlighted the vulnerability of the fourth intercostal nerve during axillary dissection [3], while a surgeon survey reported low adoption of the transaxillary approach (<10%), with changes in nipple sensation cited as one of the most common early complications of augmentation overall, though not stratified by incision [41].

In summary, direct evidence for NAC sensory outcomes after transaxillary augmentation remains limited. Small clinical series report generally preserved sensation [24] but lack objective testing. Anatomic data strongly support the plausibility of nerve injury at this site [3, 42].

#### Transumbilical Incisions

The transumbilical breast augmentation (TUBA) approach was addressed in two large retrospective series. Both consistently reported a negligible risk of NAC sensory loss, reflecting the absence of a breast skin incision. Across cohorts totaling nearly 3,000 patients, normal nipple sensation was consistently maintained following augmentation, regardless of whether implants were placed in subglandular or subpectoral planes. However, outcomes in the larger series were based on chart review without objective testing, and detailed quantitative outcomes were not reported in the smaller cohort. [37, 38]

#### Other Incision Variants

Several studies explored less common modifications of standard approaches, including perinipple broken-line variations and medial periareolar access. A perinipple broken-line technique in ten subfascial augmentations reported no subjective changes in NAC sensation at 12 months, although the absence of objective testing and the small sample size limit conclusions [22]. A medial periareolar variant described in a five-patient series similarly showed mostly preserved sensation, with one transient postoperative reduction and one case of mild persistent hypoesthesia at eight months [27]. In summary, these niche modifications appear to preserve NAC function in small, subjective series, but the data remain insufficient for form conclusions.

Across incision types, reported sensory outcomes varied, but methodological heterogeneity limited direct comparison. A comparative synthesis is presented in the discussion section.

## Discussion

This scoping review synthesized 37 studies addressing nipple–areola complex (NAC) sensory outcomes following breast augmentation across different incision types. Several consistent trends emerged, though heterogeneity in study design, follow-up duration, and outcome assessment limits the strength of direct comparisons.

### Predominance of Inframammary and Periareolar Incisions

The published evidence is heavily weighted toward inframammary and periareolar approaches, which together accounted for nearly two-thirds of included studies. This emphasis parallels real-world surgical practice, where inframammary incisions are consistently the most widely used worldwide. A survey of American Society for Aesthetic Plastic Surgery members found that 64% of surgeons preferred inframammary access compared with 25% for periareolar and fewer than 10% for axillary or transumbilical approaches [41]. Analysis of over 11,000 breast augmentations reported to the American Board of Plastic Surgery Maintenance of Certification Tracer Database between 2011 and 2015 confirmed this dominance, with inframammary incisions used in 75.1% of cases compared to 17.8% periareolar and 4.1% transaxillary [40]. These findings confirm that inframammary access is both the most studied and most practiced technique, while evidence for alternative incisions remains sparse and conclusions for those approaches therefore more tentative.

### Methodological Heterogeneity and Assessment Bias

A striking feature across the literature is the predominance of subjective sensory assessment. Fifty-four percent of included studies relied solely on patient self-report and observation, while only five employed exclusively objective tools such as Semmes–Weinstein monofilaments or pressure-specified sensory devices. Prospective cohorts more often incorporated objective or mixed methods, yet standardized testing was inconsistently applied, and many retrospective series relied on chart review or surgeon recall without structured assessment. This methodological imbalance complicates interpretation. Although subjective reporting captures patient-centered outcomes, it is vulnerable to recall bias, underreporting of transient symptoms, and wide interindividual variability [45]. Objective tools, although more precise, were adopted far less frequently and often without consistent protocols, limiting cross-study comparability. Future investigations must prioritize standardized, validated measures to generate reliable, comparable data. In keeping with the scoping review methodology, this synthesis highlights domains of variability and gaps rather than formally assessing bias, underscoring the need for more standardized approaches in future research.

### Incision Type in Context of Modifying Factors

Across all approaches, incision type alone rarely determined long-term sensory outcome. Instead, modifying factors such as implant-to-breast volume ratio, implant plane, and incision placement within a given fold, consistently emerged as critical. Larger implants in smaller breasts increased the risk of hypoesthesia regardless of incision, while dual-plane and subfascial placements often preserved sensation more effectively than purely subglandular pockets [19, 32]. Similarly, central inframammary incisions were associated with greater loss of inferior quadrant sensitivity compared with lateralized placements [20]. These findings underscore that incision choice interacts with broader surgical decisions, and isolated comparisons of incision type may obscure the true determinants of NAC function.

### Less Common Approaches

Evidence for transareolar, transaxillary, and transumbilical access remains sparse and methodologically weaker. Transareolar incisions demonstrated a consistent discordance between objective and subjective outcomes: measurable long-term deficits on sensory testing contrasted with relatively few patients reporting functional changes [28]. Transaxillary series generally suggested preserved sensation, but cadaveric mapping highlights the vulnerability of the fourth intercostal nerve as it traverses the axillary tunnel, establishing clear anatomical risk [39, 42]. By avoiding direct breast incisions, TUBA consistently showed negligible risk of NAC dysfunction, though its relevance is restricted by reliance on saline devices and declining modern use [37, 46]. Thus, while IMF and periareolar approaches remain the most reliable access points, outcomes for less common incisions must be interpreted with caution, as sample sizes are small and designs are often retrospective.

### Clinical Implications

For surgical practice, several key messages arise. First, patients can be reassured that permanent sensory loss is rare across all incision types, though temporary numbness is common in the early postoperative period. Second, relative differences exist: Periareolar incisions are associated with higher risk in large retrospective datasets [23], while lateralized IMF access offers the most consistently favorable profile [ 20]. Third, risk is strongly modified by factors under surgical control including implant size, plane, and incision placement, highlighting the need for tailored preoperative planning. Finally, the frequent mismatch between patient-perceived and objectively measured outcomes reinforces the value of incorporating both perspectives in counseling and follow-up.

### Research Priorities

Future studies should address several gaps. Standardized, validated sensory testing ideally combining objective modalities with patient-reported outcomes, must be incorporated into prospective multicenter cohorts. Comparative studies should account for confounders such as implant volume, plane of placement, and surgical technique, which frequently overshadow incision type in predicting outcomes. Finally, high-quality data on less common approaches, particularly transaxillary and transareolar, remain urgently needed to guide evidence-based practice.

## Limitations

This scoping review has several limitations. First, the included studies span more than two decades (2000–2025), during which surgical practice, implant technology, and incision preferences evolved substantially. Earlier cohorts predominantly reported periareolar and inframammary approaches, while transaxillary and transumbilical techniques appeared more frequently in later years. Therefore, differences in sensory outcomes may partially reflect temporal changes rather than incision type alone. Second, methodological heterogeneity, particularly wide variation in sensory assessment tools, follow-up duration, and definitions of “sensory change” limits direct comparison between studies. Third, systematic and narrative reviews were included as sources of evidence to map study designs and topics, however, because these reviews summarize primary studies also included in our dataset, descriptive characteristics such as incision distributions, follow-up durations, sensory assessment methods, and geographic patterns may reflect overlapping data. To avoid duplication, review-level patient counts and outcomes were not pooled with primary cohorts. Fourth, most included studies relied heavily on subjective patient reporting, with relatively few using standardized objective testing, which may introduce reporting bias. Finally, as a scoping review, this study aims to map concepts and variability rather than quantitatively pool outcomes or assess risk of bias, and conclusions should therefore be interpreted descriptively.

## Conclusion

This review demonstrates that while incision choice influences sensory outcomes, persistent deficits are uncommon and rarely disabling. Inframammary access, particularly when lateralized, remains the most favorable and widely adopted approach. Periareolar incisions may confer higher relative risk but with low absolute incidence of long-term dysfunction. Transareolar, transaxillary, and transumbilical techniques offer limited or inconsistent evidence, warranting caution in interpretation. Future progress will depend on rigorous, standardized, and adequately powered studies that reflect both objective sensory testing and patient-centered outcomes.

## Supplementary Information

Below is the link to the electronic supplementary material.

Supplementary file1 (DOCX 30 KB)

Supplementary file2 (XLSX 14 KB)
