# Supplementary material for: Comparing Nipple and Areola Sensory Outcomes and Nerve-Related Complications Using Different Incision Types in Breast Augmentation: A Scoping Review
Source: Aesthetic Plast Surg. 2026 Feb 18;50(12):4697–707. doi: 10.1007/s00266-026-05681-x (PMC13315334; doi:10.1007/s00266-026-05681-x)
Supplement: Supplementary file 1 — Supplementary file1 (DOCX 30 KB) [file 266_2026_5681_MOESM1_ESM.docx]

Complete Search Strategy

# Date of Search

2025-07-31

# Databases for Search

PubMed (via NLM), Embase (via Elsevier), Scopus (Elsevier), Cochrane Library (Wiley), Google Scholar (for supplementary searching).

# Final Comprehensive Search Strategy (All Fields)

("breast augmentation" OR "breast implants" OR augmentation OR mammaplasty OR mammoplasty OR "breast enlargement" OR "cosmetic breast surgery") AND
(nipple OR areola OR "nipple-areola complex" OR "nipple-areolar complex" OR NAC OR "nipple area" OR "areolar region") AND
(sensation OR sensory OR sensitivity OR numbness OR paresthesia OR desensitization OR hyposensitivity OR hypersensitivity OR altered sensation OR "sensory loss" OR "nerve injury" OR "nerve damage" OR neuropathy OR "sensory dysfunction") AND
(incision OR incisions OR "surgical approach" OR technique OR "operative technique" OR periareolar OR transareolar OR inframammary OR transaxillary OR axillary OR transumbilical OR umbilical OR "surgical access" OR "incision site")

# Language and Filters

Searches were limited to English-language studies published from 2000 to 2025. Where available, filters for human subjects and relevant publication types (e.g., clinical studies, reviews, scoping reviews) were applied.

# Search Methodology Notes

The search strategy was developed according to PRISMA-ScR and JBI guidelines. Both controlled vocabulary (MeSH, Emtree) and free-text keywords were combined to maximize sensitivity. The strategy was piloted in PubMed, adapted for each database. All records were exported, de-duplicated, and screened against predefined inclusion and exclusion criteria.

Cochrane Library:

("breast augmentation" OR "breast implants" OR augmentation OR mammaplasty OR mammoplasty OR "breast enlargement" OR "cosmetic breast surgery") AND
(nipple OR areola OR "nipple-areola complex" OR "nipple-areolar complex" OR NAC OR "nipple area" OR "areolar region") AND
(sensation OR sensory OR sensitivity OR numbness OR paresthesia OR desensitization OR hyposensitivity OR hypersensitivity OR altered sensation OR "sensory loss" OR "nerve injury" OR "nerve damage" OR neuropathy OR "sensory dysfunction") AND
(incision OR incisions OR "surgical approach" OR technique OR "operative technique" OR periareolar OR transareolar OR inframammary OR transaxillary OR axillary OR transumbilical OR umbilical OR "surgical access" OR "incision site")

Filter: 01/01/2000 – 31/07/2025

Embase:

("breast augmentation" OR "breast implants" OR augmentation OR mammaplasty OR mammoplasty OR "breast enlargement" OR "cosmetic breast surgery") AND
(nipple OR areola OR "nipple-areola complex" OR "nipple-areolar complex" OR NAC OR "nipple area" OR "areolar region") AND
(sensation OR sensory OR sensitivity OR numbness OR paresthesia OR desensitization OR hyposensitivity OR hypersensitivity OR altered sensation OR "sensory loss" OR "nerve injury" OR "nerve damage" OR neuropathy OR "sensory dysfunction") AND
(incision OR incisions OR "surgical approach" OR technique OR "operative technique" OR periareolar OR transareolar OR inframammary OR transaxillary OR axillary OR transumbilical OR umbilical OR "surgical access" OR "incision site")

AND (2000:py OR 2001:py OR 2002:py OR 2003:py OR 2004:py OR 2005:py OR 2006:py OR 2007:py OR 2008:py OR 2009:py OR 2010:py OR 2011:py OR 2012:py OR 2013:py OR 2014:py OR 2015:py OR 2016:py OR 2017:py OR 2018:py OR 2019:py OR 2020:py OR 2021:py OR 2022:py OR 2023:py OR 2024:py OR 2025:py) AND [female]/lim AND ('article'/it OR 'clinical trial'/it OR 'review'/it)

Scopus:

TITLE-ABS-KEY ( ( "breast augmentation" OR "breast implants" OR augmentation OR mammaplasty OR mammoplasty OR "breast enlargement" OR "cosmetic breast surgery" ) AND ( nipple OR areola OR "nipple-areola complex" OR "nipple-areolar complex" OR NAC OR "nipple area" OR "areolar region" ) AND ( sensation OR sensory OR sensitivity OR numbness OR paresthesia OR desensitization OR hyposensitivity OR hypersensitivity OR "altered sensation" OR "sensory loss" OR "nerve injury" OR "nerve damage" OR neuropathy OR "sensory dysfunction" ) AND ( incision OR incisions OR "surgical approach" OR technique OR "operative technique" OR periareolar OR transareolar OR inframammary OR transaxillary OR axillary OR transumbilical OR umbilical OR "surgical access" OR "incision site" ) ) AND PUBYEAR > 1999 AND PUBYEAR < 2026 AND ( LIMIT-TO ( SUBJAREA , "MEDI" ) ) AND ( LIMIT-TO ( DOCTYPE , "ar" ) OR LIMIT-TO ( DOCTYPE , "re" ) ) AND ( LIMIT-TO ( LANGUAGE , "English" ) )

PubMed:

**((breast augmentation) OR (breast implants) OR (augmentation) OR (mammaplasty) OR (mammoplasty) OR (breast enlargement) OR (cosmetic breast surgery)) AND ((nipple) OR (areola) OR (nipple-areola complex) OR (nipple-areolar complex) OR (nipple area) OR (areolar region)) AND ((sensation) OR (sensory) OR (sensitivity) OR (numbness) OR (paresthesia) OR (desensitization) OR (hyposensitivity) OR (hypersensitivity) OR (altered sensation) OR (sensory loss) OR (nerve injury) OR (nerve damage) OR (neuropathy) OR (sensory dysfunction)) AND ((incision) OR (incisions) OR (surgical approach) OR (technique) OR (operative technique) OR (periareolar) OR (transareolar) OR (inframammary) OR (transaxillary) OR (axillary) OR (transumbilical) OR (umbilical) OR (surgical access) OR (incision site)) Filters: Adaptive Clinical Trial, Case Reports, Clinical Study, Clinical Trial, Comparative Study, Controlled Clinical Trial, Corrected and Republished Article, Dataset, Equivalence Trial, Evaluation Study, Meta-Analysis, Multicenter Study, Observational Study, Randomized Controlled Trial, Review, Scoping Review, Systematic Review, Technical Report, Validation Study, English, Humans, Female, Exclude preprints, from 2000/1/1 - 2025/7/31**
